# Functionalized hydrogel sequentially deliver tannic acid and bioactive probiotics for radiation-induced skin injury

**DOI:** 10.1016/j.mtbio.2025.102753

**Published:** 2025-12-31

**Authors:** Xiaowen Han, Chen Zhou, Ruiling Xu, Zhimin Jia, Ying Liu, Shan Chen, Wei Tang, Xiaoan Li, Liangxue Zhou, Yong Sun

**Affiliations:** a NHC Key Laboratory of Nuclear Technology Medical Transformation, Mianyang Central Hospital, School of Medicine, University of Electronic Science and Technology of China, Mianyang, 621000, China; b National Engineering Research Center for Biomaterials, Sichuan University, 29 Wangjiang Road, Chengdu, Sichuan, 610064, China; c Animal Disease Prevention and Green Development Key Laboratory of Sichuan Province, College of Life Sciences, Sichuan University, 29 Wangjiang Road, Chengdu, Sichuan, 610064, China; d Department of Nuclear Medicine, Mianyang Central Hospital, Mianyang, China; e Institute of Materials, China Academy of Engineering Physics, Jiangyou, 621907, China; f Sichuan Provincial Engineering Research Center of Nuclear Medical Equipment Translation and Application, Mianyang Central Hospital, School of Medicine, University of Electronic Science and Technology of China, Mianyang, 621000, China

**Keywords:** Radiation-induced skin injury (RISI), Temporally programmed delivery, Functionalized hydrogel, Hierarchical therapeutic strategy

## Abstract

As skin injuries caused by radiotherapy can significantly impede the healing process, it is essential to eliminate the interference of excessive reactive oxygen species (ROS) in the treatment of radiation-induced skin injury (RISI). To address this, we developed a temporally programmed hydrogel designed to enhance RISI repair, which was synthesized through the assembly of *Lactobacillus reuteri* (*L. reuteri*) and tannic acid (TA)-loaded hydrogel (Gel/LT). These hydrogels demonstrated satisfactory free radical scavenging capacity in the early stages, achieving an effective clearance rate of 93.3 %, thereby reducing the production of ROS associated with RISI. Furthermore, the *L. reuteri* encapsulated by metal-polyphenol self-assembly is released at the wound site in response to the wound microenvironment, promoting angiogenesis and tissue regeneration. Both *in vitro* and *in vivo* experiments demonstrated that Gel/LT achieved ROS scavenging and tissue repair effects at different stages of RISI. The transcriptome results indicate that these hydrogels facilitate a transition from an immune response to cellular proliferation by reducing oxidative stress and upregulating the expression of anti-inflammatory genes. Additionally, they promote the regeneration of extracellular matrix components, such as collagen, ultimately achieving superior repair efficacy compared to the commercial drug amifostine. This hierarchical therapeutic strategy permits temporal drug delivery at various stages of the repair process, presenting a novel approach for the treatment of RISI.

## Highlights

• A temporal programmed hydrogel specifically for RISI repair was constructed by integrating the assembly of *Lactobacillus reuteri* (*L. reuteri*) and tannic acid (TA).
• The hydrogel exhibited the timing regulation through responses to the microenvironment of RISI.
• The hydrogel facilitates the transition from the immune phase to the proliferation phase by alleviating oxidative stress and inflammatory responses.

## Graphical abstract

**Figure:**
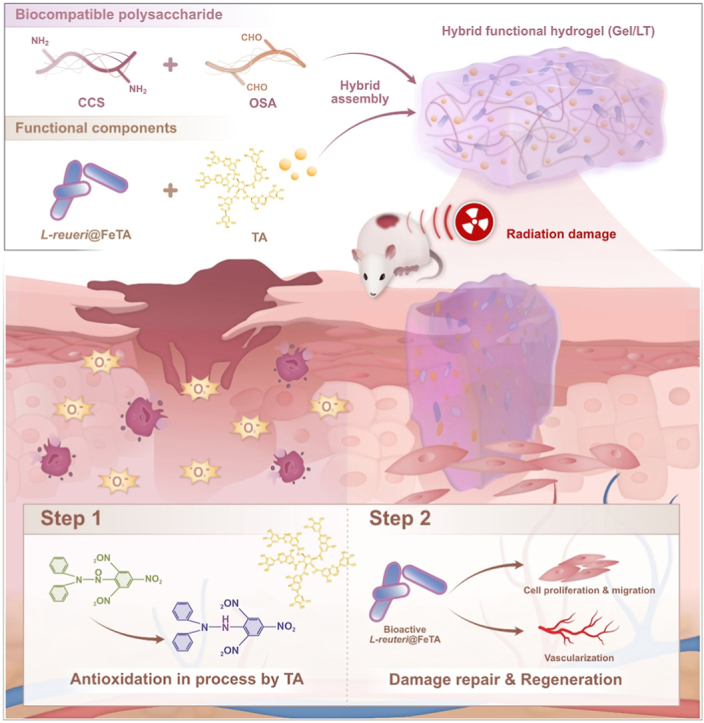

## 1 Introduction

As the body's primary line of defense against external stimuli, the skin is particularly susceptible to radiation-induced damage, which can occur in radiation-related engineering projects and nuclear accidents, such as clinical external beam radiotherapy [[1], [2], [3]]. Under the influence of radiation, a significant accumulation of reactive oxygen species (ROS) typically occurs at the wound site, accompanied by damage to surrounding normal cells and blood vessels, thereby complicating spontaneous wound healing [[4], [5], [6]]. In the early stages of injury, ionizing radiation directly induces double-strand breaks in cellular DNA and triggers excessive generation of ROS, leading to necrosis [[7], [8], [9]]. Furthermore, sustained oxidative stress impairs cellular functions and establishes a pro-inflammatory microenvironment at the wound site, which further promotes ROS production and ultimately hinders angiogenesis and tissue repair [[10], [11], [12]]. Therefore, a hierarchical therapeutic strategy that first eliminates ROS, followed by tissue repair, is essential for the treatment of radiation-induced skin injury (RISI).

Living probiotics may play a crucial role in regulating and alleviating various complex pathological symptoms in humans by continuously releasing bioactive substances such as organic acids, bacteriocins, and enzymes, which also without inducing additional inflammatory responses [[13], [14a], [14b], [15], [16]]. In contrast to novel therapeutic agents, such as growth factors and antioxidants, probiotics can consistently release bioactive substances without being hindered by the depletion of raw materials during therapy [17,18]. These functional and structural advantages endow probiotics with significant potential for the treatment of RISI [19]. However, it remains a challenge to enhance the antioxidant capacity and viability of probiotics in the harsh wound microenvironment, which is necessary for implementing a RISI-specific hierarchical therapeutic approach.

Due to its abundant hydroxyl groups, tannic acid (TA) exhibits satisfactory antioxidant capacity and has been widely utilized in the development of antioxidant materials [[20], [21], [22]]. Furthermore, TA can chelate with metal particles to construct a "nano-coating" supramolecular structure through layer-by-layer assembly [[23], [24], [25]]. This supramolecular structure can respond to changes in pH within its microenvironment to adjust the complexation strength between the chelates, thereby achieving a hierarchical response at different stages. Recent studies have demonstrated that this nanostructure can be stably loaded onto the surface of strains and effectively regulate the release behavior of strains under varying pH conditions, facilitating phased mediation in response to different stages of the microenvironment [26].

Hydrogels that possess a microstructure analogous to that of the extracellular matrix (ECM) demonstrate exceptional biocompatibility and have the potential to act as effective carriers for small-molecule drugs [[27], [28], [29], [30], [31], [32]]. Building on this premise, this study proposes a hierarchical treatment strategy that integrates surface nano-probiotics and TA within hydrogels, thereby promoting the sequential healing of RISI. The components of TA demonstrate an effective free radical-scavenging capability in the early stages, which reduces cell necrosis and inflammation, thereby preventing tissue deterioration. Subsequently, the living strains are released in response to the microenvironment and participate in the processes of tissue regeneration and vascularization. Transcriptome and *in vivo* results indicate that hydrogels can effectively exert a variety of bioactivities, including the removal of excessive ROS, reduction of inflammation, and repair of necrotic cells and blood vessels at various stages of RISI healing. This approach presents a novel strategy for the treatment of radiation injuries.

## 2 Materials and methods

### 2.1 Materials

Carboxymethyl chitosan with degree of carboxylation ≥80 % (CCS) and sodium alginate (SA) were sourced from Bloomage Freda Biopharm Corporation in Shandong, China. *Lactobacillus reuteri* (*L. reuteri*) was obtained from the China General Microbiological Culture Collection Center (CGMCC). Sodium periodate (NaIO_4_) was procured from Macklin (Shanghai, China). Ferric chloride (FeCl_3_) and TA were acquired from Aladdin Bio-Chem Technology Corporation (Shanghai, China). DeMan-Rogosa-Sharpe (MRS) medium, phosphate-buffered saline (PBS), cell counting kit-8 (CCK-8), Dulbecco's modified Eagle medium (DMEM), fetal bovine serum, penicillin-streptomycin, and trypsin were purchased from Gibco (USA). Alginate lyase (≥10,000 units/g) was sourced from Sigma-Aldrich Corporation (USA). Additionally, 4′,6-diamidino-2-phenylindole (DAPI), DCFH-DA, propidium iodide (PI), fluorescein diacetate (FDA), and a modified Masson's trichrome stain kit were obtained from Beijing Solarbio Science & Technology Co., Ltd. Matrigel was obtained from Corning Incorporated (USA). ABTS and DPPH free radical scavenging ability assay kits were purchased from Beijing Boxbio Co., Ltd. The DNA damage assay kit, utilizing γ-H2AX immunofluorescence and paraformaldehyde, was supplied by Beyotime Biotechnology Co., Ltd. Gentian violet was acquired from Biosharp Technology Co., Ltd.

### 2.2 Preparation and characterization of Gel/LT

The Gel/LT hydrogel was successfully prepared based on a hierarchical assembly strategy, as described in our previous study with some modifications [25,33]. Briefly, *L. reuteri* were first suspended in PBS and vortexed with FeCl_3_ (1.25 mg/mL) and a TA solution (5 mg/mL), resulting in the formation of *L. reuteri*@FeTA. In this research, *L. reuteri* was cultured using MRS medium. To obtain the OSA, SA was completely dissolved and reacted with NaIO_4_ (w:w = 1:1) while being stirred for 12 h, and ethylene glycol was used to terminate the oxidation reaction. The acquired product was dialyzed and subsequently freeze-dried. The Gel hydrogel was synthesized via the Schiff base reaction between CCS and OSA, as described in the literature [34]. To obtain the Gel/LT hydrogel, a mixture of 10^6^ CFU/mL *L. reuteri*@FeTA and 1 mg/mL TA solution was combined with the OSA solution, followed by gelation with CCS. The Gel/TA and Gel/L hydrogels were prepared using the same method, incorporating either TA solution or *L. reuteri*@FeTA.

### 2.3 Physicochemical properties characterization

The chemical constitution of the copolymers or hydrogels was recorded using an ATR-FTIR spectrometer (Nicolet 6700, Thermo Electron Corporation, USA). The morphology, pore size, equilibrium swelling ratio, *in vitro* degradation ratio, storage modulus (G′), and loss modulus (G″) of the hydrogels were measured in accordance with our recent literature [24]. The adhesive property of hydrogel had been measured by universal testing machine (CMT2103, METS, USA). The released rate of TA and lactic acid in hydrogel were determined by colorimetric method of the OD value under PBS and alginate hydrolysis environments. Each test included at least three samples.

### 2.4 Cell and animal

Embryonic fibroblast cell lines (NIH3T3) were cultured in DMEM supplemented with 10 % fetal bovine serum and 1 % penicillin/streptomycin. Human umbilical vein endothelial cells (HUVECs) were obtained from Xiamen Immocell Biotechnology and maintained at 37 °C in a 5 % CO_2_ atmosphere. Specific pathogen-free (SPF) male BALB/c mice, weighing approximately 25 g, were acquired from GemPharmatech Co., Ltd. The mice were housed in a dedicated SPF animal facility, where they had ad libitum access to water and food, were maintained under a regular light-dark cycle, and were kept in an SPF-compliant living environment. All animal-related procedures in this study adhered to the 3Rs principle (Replacement, Reduction, and Refinement) for the use of experimental animals and received approval from the Animal Ethics Committee of Mianyang Central Hospital.

### 2.5 Cell viability

First, 500 μL of hydrogel samples were immersed in 5 mL of DMEM and incubated at 37 °C for 72 h. The resulting extraction solution was subsequently filtered through a 0.22-μm membrane filter to eliminate potential bacterial contamination, after which it was supplemented with 10 % FBS. 3T3 or HUVEC cells were then seeded in a 24-well plate at a density of 2 × 10^4^ cells per well and incubated for 24 h, followed by a co-incubation period of 48 h with the extraction solutions of Gel, Gel/TA, Gel/L, and Gel/LT, respectively. Cells seeded in a 96-well plate without extraction solutions served as the positive control. The culture medium was carefully discarded, and cell viability was appraised using the CCK-8 assay. Live and dead NIH3T3 cells were observed using a confocal laser scanning microscope (CLSM Stellaris 5, Leica, Germany) after staining with FDA/PI solution in the dark.

### 2.6 Intracellular ROS and DNA damage detection

NIH3T3 cells were seeded in confocal dishes at a density of 5 × 10^4^ cells per dish. Twenty-four hours prior to radiation exposure, the culture medium was replaced with the respective extraction solutions. Subsequently, the cells were stained with DCFH-DA solution for 30 min, after which the staining solution was removed. The cells were then exposed to 6 Gy of X-ray radiation (X-Rad 320, Precision Xray Inc., USA) at a constant dose rate of 2 Gy/min. Finally, DCFH-DA and γH2AX dyes were employed to assess ROS levels and DNA damage, which were observed using CLSM. The statistical data of ROS fluorescence intensity and the number of DNA damage foci were analyzed with ImageJ software.

### 2.7 Tube formation *in vitro*

100 μL of Matrigel was evenly distributed across the bottom of a 24-well plate and allowed to polymerize. Irradiated HUVECs were seeded into the wells at a density of 5 × 10^4^ cells per well and cultured with conditioned media. After 8 h, tube formation was assessed using both a microscope and CLSM. Cell viability was appraised using the CCK-8 assay. The tube length and the number of major junctions were quantified using ImageJ software.

### 2.8 Transwell assay

In the transwell migration assay, NIH3T3 cells exposed to 6 Gy of X-ray radiation were seeded in the upper chamber of 24-well transwell plates at a density of 1 × 10^4^ cells per well and cultured in DMEM. Extraction solutions were added to the lower chamber, followed by a 24-h incubation period. Subsequently, the cells remaining in the upper chamber were fixed with 4 % paraformaldehyde and stained with gentian violet. Photographs of the migrated cells were captured using a microscope.

### 2.9 Murine radiation induced skin injury *in vivo*

Balb/c mice were randomly assigned to six groups, each receiving a single dose of 20 Gy X-ray irradiation to the thigh at a rate of 2 Gy/min [35]. Fourteen days post-irradiation, hair loss commenced at the irradiated site, indicating successful radiation-induced damage [36]. At this juncture, various hydrogels were applied to the irradiated sites for treatment, with saline and amifostine serving as negative and positive controls, respectively. The wound sites were photographed on days 4, 7, and 14 following the initiation of treatment, and the skin damage at these sites was assessed according to the RTOG criteria. On days 7 and 14, skin tissues were gathered, fixed, and embedded in paraffin or frozen, sliced, and subsequently stained with hematoxylin and eosin (H&E) as well as Masson's Trichrome Staining. Immunofluorescence staining for ROS, TNF-α, CD31, CD86, and CD206 was performed to observe the wound repair process.

### 2.10 Biosafety of hydrogel

For the biosafety assessment of hydrogels, Balb/c mice were stochastically divided into five groups (*n* = 3). A total of 100 μL of hydrogel precursor solution was injected into the dorsal region of the mice, with the PBS group serving as the control. On day 14, blood samples were collected from the mice for hematological analysis, and major organs (including the heart, liver, spleen, kidneys, and lungs) were harvested for tissue embedding, sectioning, and H&E staining.

### 2.11 RNA sequencing

Transcriptome analysis of irradiated skin samples was conducted by Majorbio. Initially, total RNA was isolated using Trizol Reagent in accordance with the manufacturer's instructions, and subsequently quantified using a NanoDrop2000 (Thermo Fisher Scientific). Following RNA purification and reverse transcription, the RNA-seq transcriptome library was prepared. Library construction was performed according to the Illumina® Stranded mRNA Prep Ligation protocol from Illumina. Libraries were subjected to size selection for 300 bp cDNA target fragments using 2 % Low Range Ultra Agarose gel, followed by PCR amplification with Phusion DNA polymerase (New England Biolabs, NEB) for 15 cycles. The raw paired-end reads were trimmed and quality-filtered using fastp software with default parameters. Subsequently, the clean reads were individually aligned to the reference genome in orientation mode via HISAT2 software. All subsequent downstream analyses were conducted using high-quality clean data. Differential expression analysis between the two comparison groups was conducted using the DESeq2 R package. Genes were classified as significantly differentially expressed genes (DEGs) if they satisfied the criteria of |log_2_ fold change (FC)| ≥ 1 and a false discovery rate (FDR) ≤ 0.05, as determined by DESeq2. Additionally, functional enrichment analyses, including Gene Ontology (GO) and Kyoto Encyclopedia of Genes and Genomes (KEGG) pathway analyses, were conducted to identify DEGs that were prominently enriched in specific GO terms and metabolic pathways. These enrichments were deemed statistically significant when the Bonferroni-corrected P-value was less than or equal to 0.05, using the entire transcriptome as the background reference. Finally, representative gene expression of TNF-α、IL-4、IL-6、Col1 and Col3 was evaluated via q-PCR technique as mentioned above.

### 2.12 Statistical analysis

All data were presented as mean ± standard deviation (SD) of at least three representative experiments. Statistical analysis was performed using Prism software (GraphPad Software Inc.) by Student's t-test (unpaired and two-tailed), one-way ANOVA. The significance level assumed at *p* < 0.05 (∗), *p* < 0.01 (∗∗), *p* < 0.001 (∗∗∗), *p* < 0.0001 (∗∗∗∗).

## 3 Results and discussion

### 3.1 Synthesis and structural characterization of hydrogels

**Scheme. 1:**
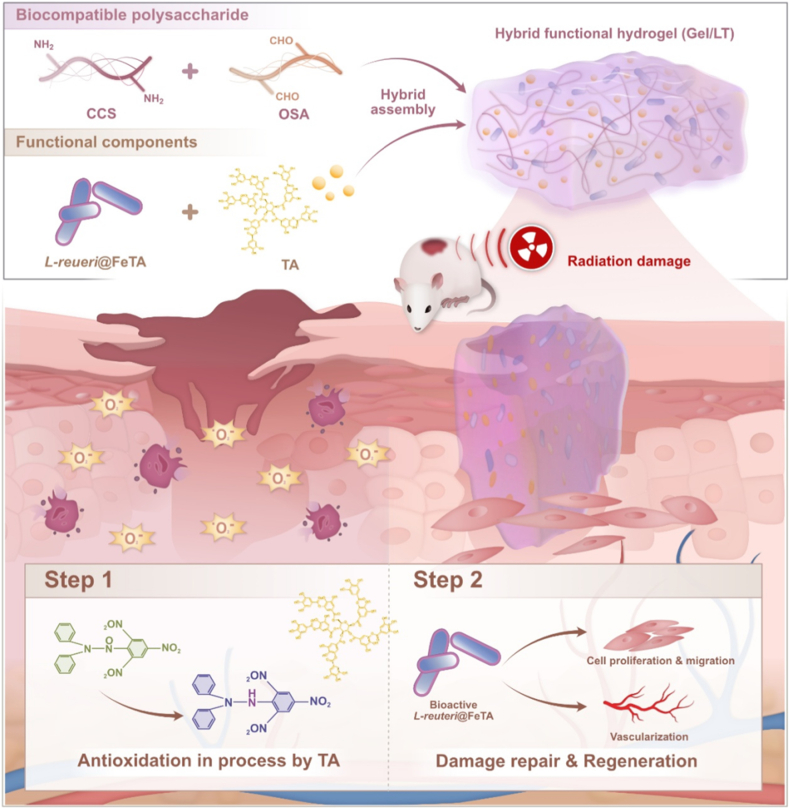
The synthesis process and graded treatment schematic of hydrogels by sequential response microenvironment of RISI.

**Fig. 1:**
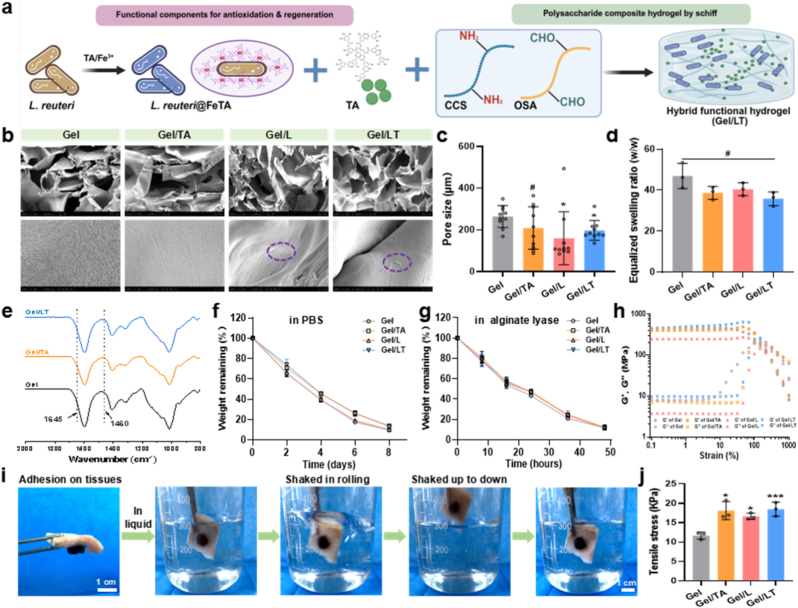
Preparation and characterization of Gel/LT. (a) The synthesis scheme process of hydrogels; (b) Representative SEM images of each hydrogel; Pore size (c) and equalized swelling ratio (d) of hydrogels; (e) The ATR-FTIR spectrum of hydrogels; *In vitro* degradation ratio of hydrogels in PBS (f) and alginate lyase (g); (h) The strain sweep test of each hydrogel; (i) Tissue adhesion properties of hydrogels; (j) The tensile stress of hydrogels in tissue adhesion. ^#^p > 0.05, ∗p < 0.05, ∗∗∗p < 0.001.

The synthesis schematic diagram of hydrogels is presented in Scheme 1. First, *L. reuteri* was encapsulated with a nano-coating through layer-by-layer self-assembly involving TA and Fe^3+^. SEM and TEM images confirmed the successful formation of this coating on the surface of *L. reuteri* (Fig. S1). This supramolecular structure was able to maintain the activity of the strains, thereby enhancing their biological properties [24]. As a precursor of hydrogels, OSA polymer was synthesized through the oxidation of sodium periodate. The peak observed at 1730 cm^−1^ in the ATR-FTIR spectrum further confirmed the presence of an aldehyde group [37] (Fig. S2). Subsequently, the Gel/LT hydrogel was synthesized using a one-pot method (Fig. 1a). The SEM images revealed that each hydrogel exhibits a typical porous structure, with *L. reuteri* uniformly distributed throughout the hydrogel network (Fig. 1b). TEM images also confirmed that the *L. reuteri*@FeTA could stably present in the hydrogel (Fig. S3). The average pore size of hydrogel had been decreased after loading with *L. reuteri*@FeTA, that may due to the effect of *L. reuteri* to cross-linked the molecular chains [25] (Fig. 1c). In addition, the swelling ratio of hydrogel was not affected significantly after loading with *L. reuteri*@FeTA and TA (Fig. 1d). The ATR-FTIR spectrum (Fig. 1e) confirmed that the hydrogels were successfully synthesized via a Schiff-base reaction, as indicated by the characteristic peak at 1645 cm^−1^. Following the incorporation of TA and *L. reuteri*, the hydrogels demonstrated stable degradation behavior both in PBS and alginate lyase, which adapt the requirement of long-period treatment [38,39] (Fig. 1f and g). The G curves overlapped strain had not been influenced after modification, which indicated the mechanical properties of the hydrogels remained unaffected, as shown in our previous study [25] (Fig. 1h), fulfilling the property requirements for wound dressing materials. Meanwhile, the hydrogels demonstrated stable adhesion to skin tissue, even in a fluid environment (Fig. 1i and multimedia component 1), and the tensile stress had been enhanced after modification (Fig. 1j). The adhesive properties of the hydrogel primarily stem from Schiff base crosslinking, and the abundant tannic acid groups within the hydrogel could enhance the physical adhesion to tissue surfaces (including hydrogen bonding, cation-π, and π-π interactions) [38,39]. These results indicated that Gel/LT has been effectively synthesized and possesses suitable physicochemical properties that are beneficial for the repair of injured sites.

### 3.2 Gel/LT hydrogels response to pH changes and mitigating radiation-induced damage

**Fig. 2:**
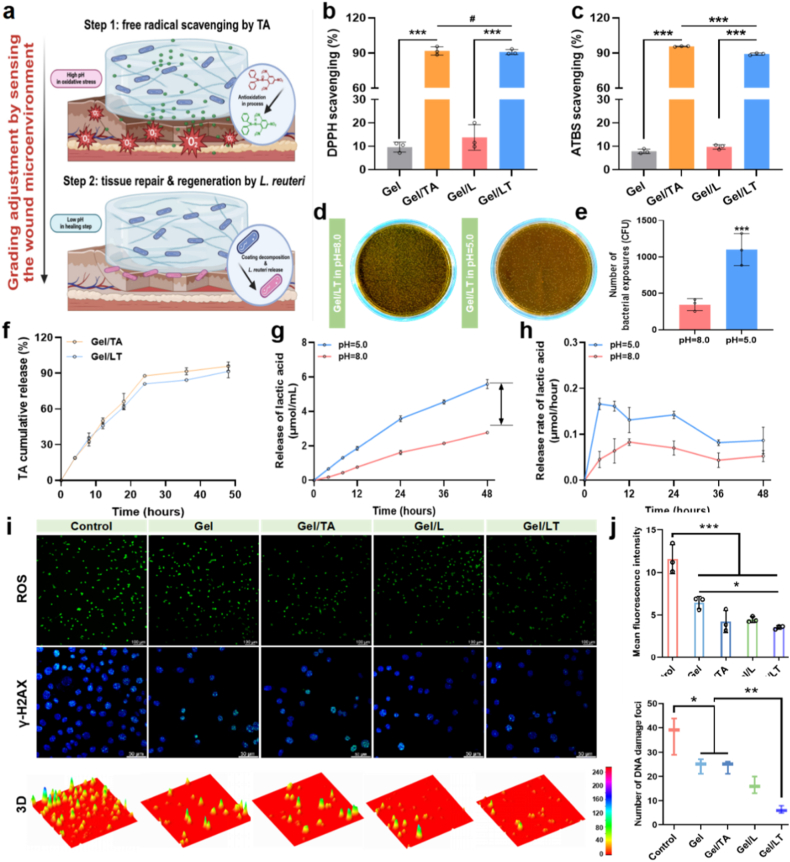
pH-responsive and radioprotective effect of Gel/LT. (a) The grading adjustment of hydrogels by responding to the microenvironment; The DPPH (b) and ATBS (c) scavenging capacity of hydrogels; (d) The exposures of strains from hydrogels in different pH value; (e) Statistics of strains exposures; (f) The release rate of TA from hydrogels. The total release (g) and release rate (h) of lactic acid from hydrogels in different pH value. Representative images (i) and quantification (j) of mean ROS fluorescence intensity and DNA damage foci. ^#^*p* > 0.05, ∗*p* < 0.05, ∗*p* < 0.01, ∗∗∗p < 0.001.

Due to the excessive accumulation of free radicals, the microenvironment at the radiation damage site was alkaline during the initial stage. Notably, relevant studies have focused on the pH variation of irradiated wounds, which provides a crucial basis for understanding the microenvironmental characteristics of RISI. Specifically, Steffen et al. monitored and reported the changes in wound pH after radiotherapy. Their findings revealed a statistically significant difference in pH values between irradiated and non-irradiated wounds, with radiation-induced wounds exhibiting a higher pH value (7.53 ± 0.26) [40]. As the healing process prolonged, the wound transitioned into the tissue regeneration phase, and the pH of the microenvironment returned to the normal range [[41], [42], [43], [44]]. Therefore, adapting to changes in the microenvironment facilitates the implementation of a phased therapeutic strategy throughout the various stages of wound healing. Based on this, Gel/LT hydrogels can achieve sequential treatment by sensing changes in wound pH (Fig. 2a). The phenolic hydroxyl groups in TA endow the Gel/LT hydrogel with a satisfactory free radical scavenging ability, with the DPPH and ABTS radical-scavenging rates exceeding 90 %, which prevents excessive ROS production and improves the microenvironment during the early stages of healing (Fig. 2b and c). As the pH value recovers, the nano-coating on the surface disintegrates by weaken of complexation reaction, as shown by weakening of iron signal (Fig. S4), and Gel/LT could release more strains to wound site at pH 5.5 (Fig. 2d and e). This phenomenon was attributed to the change in the degree of crosslinking between TA and Fe^3+^ at different pH levels [26]. The stable release rate of TA with a linear correlation was shown in hydrogel, with a notably high release rate occurring within the first 24 h.This rapid initial release enables efficient free radical scavenging during the early phase of RISI, aligning with the critical therapeutic window for mitigating oxidative stress (Fig. 2f and Fig. S5). The strain demonstrated the ability to release various bioactive components, including lactic acid, which plays a crucial role in promoting tissue regeneration during the final stages of healing. The intelligent response of the Gel/LT hydrogel to pH levels verified its feasibility in mitigating radiation damage in a time-dependent manner, which exhibited the sequential lactic acid release behavior (Fig. 2g and h). As mentioned earlier, DNA damage and excessive ROS are critical factors in RISI [1,45]. Therefore, we subsequently examined the ability of Gel/LT to reduce intracellular ROS levels and DNA damage. In Fig. 2i and j, radiation induced a substantial accumulation of intracellular ROS, as indicated by the green fluorescence observed in the control group. Compared with the control group, the intracellular ROS levels were significantly reduced after hydrogel treatment, with the Gel/LT group showing notably lower ROS levels than the Gel group, highlighting their superior ROS scavenging capabilities. Immunofluorescent staining of γH2AX, a marker for DNA double-strand breaks (DSBs), was conducted to visualize DSBs induced by irradiation. Analysis of representative images, three-dimensional reconstructed structures, and quantification of DNA damage foci revealed that Gel/LT exhibited remarkable repair-promoting activity, significantly alleviating intracellular DSBs and confirming its potent radioprotective effect. Consequently, Gel/LT effectively safeguarded DNA from ROS-mediated damage, reduced radiation-induced oxidative stress, and ultimately inhibited cellular apoptosis.

### 3.3 Gel/LT promotes cell proliferation, migration and angiogenesis *in vitro*

**Fig. 3:**
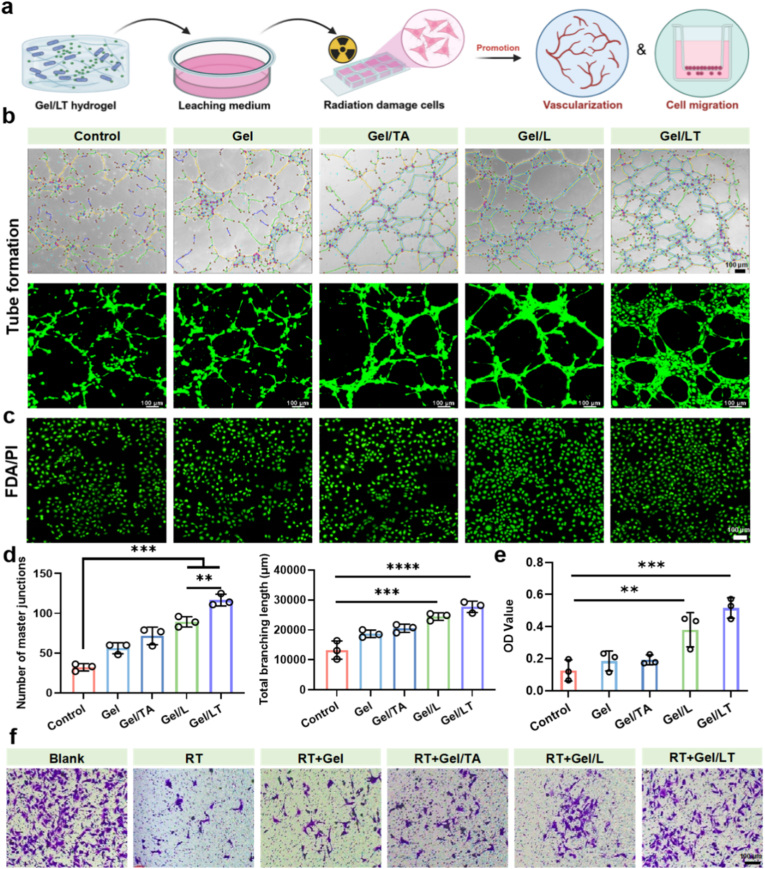
Biological function of Gel/LT. (a) The schematic diagram for transwell and tube formation experiment. Photographs of angiogenesis (b) and FDA/PI staining (c) of HUVECs under different treatments. (d) Quantification of the major junction and branching length. (e) The proliferation and (f) migration of NIH/3T3 under different treatments. ∗∗*p* < 0.01, ∗∗∗*p* < 0.001, and ∗∗∗∗*p* < 0.0001.

In RISI, cellular functions are impaired, which manifests as reduced proliferation and migration capabilities, alongside a decline in angiogenic ability, ultimately hindering tissue repair [46,47]. Consequently, the effects of Gel/LT on the proliferation and migration abilities of irradiated fibroblasts, as well as the angiogenic capacity of irradiated HUVECs, were evaluated (Fig. 3a). The live/dead staining and tube formation assay indicated that irradiated endothelial cells were unable to form tubes *in vitro*. In contrast, the hydrogel groups, particularly the Gel/L and Gel/LT hydrogel groups, exhibited excellent cytocompatibility, while also significantly enhancing the angiogenic capacity of endothelial cells, attributable to the strong tissue repair-promoting properties of probiotics. The number of major junctions and the branching length in the Gel/TA group were remarkably greater than those observed in the control group (Fig. 3b–d). Cell proliferation and migration capabilities were assessed using the CCK-8 assay and the transwell assay, respectively. As illustrated in Fig. 3e and Fig. S6, after 1 day of culture, the optical density (OD) values of fibroblasts and endothelial cells in the Gel/L and Gel/LT groups were remarkably higher than those in the other groups. This finding indicates that these two groups exhibited a stronger capacity to promote fibroblast proliferation. Subsequently, a transwell assay demonstrated variations in the migratory ability of cells under different treatments. Irradiated fibroblasts exhibited minimal migration, whereas the migration capability of cells in the hydrogel groups was slightly restored. Notably, the Gel/LT group demonstrated the highest number of migrating cells, which appeared spread and interconnected (Fig. 3f). These findings suggest that the Gel/LT hydrogel, characterized by its ROS-scavenging and probiotic-mediated repair functions, can facilitate the functional recovery of irradiated cells and enhance their proliferation, migration, and angiogenic capacities.

### 3.4 Gel/LT scavenges ROS and promotes the wound healing of RISI *in vivo*

**Fig. 4:**
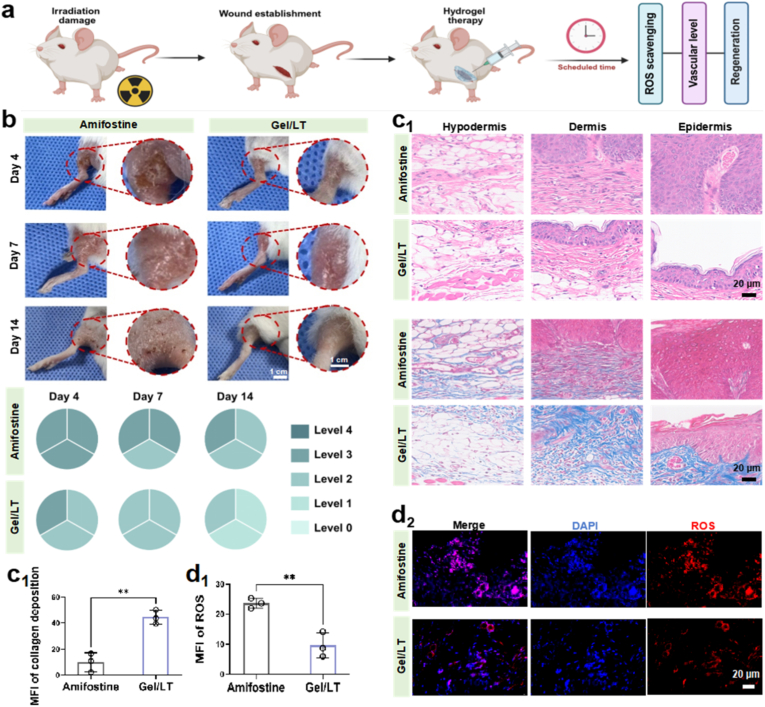
Gel/LT for murine radiation induced skin injury. (a) The schedule of radiation injury and hydrogel treatment. (b) Representative photographs and RTOG scoring of Gel/LT and amifostine. (c1) H&E and Masson staining images and (c2) Quantification of neo-collagen deposition of different treatment. (d1) Immunofluorescence images and (d2) Quantification of ROS level of different treatment at Day 7. ∗∗*p* < 0.01.

Subsequently, the biosafety of the hydrogel was investigated through subcutaneous injection. H&E staining results of major organs revealed no significant differences between the hydrogel groups and the control group, manifesting that there were no apparent toxic influences on the organ tissues of the mice following administration. Furthermore, there were no significant differences in the levels of red blood cells (RBC), white blood cells (WBC), and platelets (PLT) among the groups, suggesting the *in vivo* biosafety of the hydrogel (Fig. S7). RISI, a prevalent side effect of radiotherapy, often evolves into chronic or non-healing wounds due to the exacerbation of radiation-induced oxidative stress. The Gel/LT hydrogel's capacity to effectively scavenge ROS and promote angiogenesis prompted us to investigate its therapeutic effects on RISI. To establish the RISI model, the hind limb of mice was subjected to 20 Gy X-ray radiation to induce skin injury. As illustrated in Fig. 4a, Gel/LT hydrogels were applied to the wound site every two days, starting two weeks post-irradiation (designated as day 0). Amifostine, the only FDA-approved drug for the protection against radiation-induced mucositis and dermatitis, served as the positive control in this study. Representative images of the wounds were recorded and evaluated on days 4, 7, and 14 following treatment. Throughout the entire experimental period, the wounds in the control group exhibited extensive ulcers and necrosis, accompanied by redness, swelling, and moist desquamation. In contrast, the wounds in the amifostine group demonstrated less severe deterioration during the early stages. However, as time progressed, a certain degree of ulceration and redness continued to manifest, attributed to the low bioavailability of the drug and the insufficient tissue repair-promoting functions. Notably, the Gel/LT hydrogel, benefiting from early ROS scavenging and the robust tissue repair capacity of probiotics, not only inhibited the deterioration of the wound in its early stages—thus preventing ulceration and necrosis—but also achieved complete resolution of wound redness and swelling after 14 days, with the wound site appearing nearly indistinguishable from normal skin. The Gel/TA hydrogel, which possesses only ROS scavenging ability, alleviated wound ulceration primarily in the early stages. In contrast, the Gel/L group demonstrated inferior wound repair performance throughout the entire treatment period when compared to the Gel/LT group (Fig. 4b and Fig. S8). Skin damage was assessed using the Radiation Therapy Oncology Group (RTOG) criteria, as illustrated in the accompanying diagram. Notably, the Gel/LT hydrogel significantly reduced the severity of acute injury induced by X-ray irradiation compared to the amifostine group. H&E and Masson's trichrome staining were conducted to further appraise the effects and processes of skin repair. As illustrated in Fig. 4c and S9, the epidermis in the amifostine group exhibited severe necrosis and pathological structural defects. Two weeks post-treatment, the wound displayed hyperplastic epidermis, reduced collagen deposition, and inadequate regeneration of skin appendages. In contrast, the Gel/LT treatment group demonstrated the closest resemblance to those in the blank control group in terms of multiple key morphological indicators such as an intact epidermis, a greater number of skin appendages, and increased neo-collagen deposition after 14 days. This demonstrated that wounds treated with Gel/LT hydrogel exhibited the highest rate of tissue recovery. ROS play a crucial role in the early stages of RISI. Cells that are directly exposed to ROS experience damage to both nuclear and mitochondrial DNA, which can lead to cell death through pathways such as apoptosis and necrosis. This process is further accompanied by the release of pro-inflammatory factors, including NF-κB and IL-1, which contribute to immune cell senescence and disrupt the healing process, ultimately resulting in impaired tissue repair. Consequently, we characterized the ROS levels at the wound site on day 4 post-treatment using immunofluorescence staining. As illustrated in Fig. 4d, cells in the amifostine group exhibited abundant ROS-specific fluorescent signals, while the Gel/LT group demonstrated a significant reduction in ROS levels. This observation confirms that Gel/LT has the capacity to suppress ROS generation, thereby mitigating cellular damage and promoting tissue repair.

### 3.5 Mechanism of Gel/LT promoting RISI repair

**Fig. 5:**
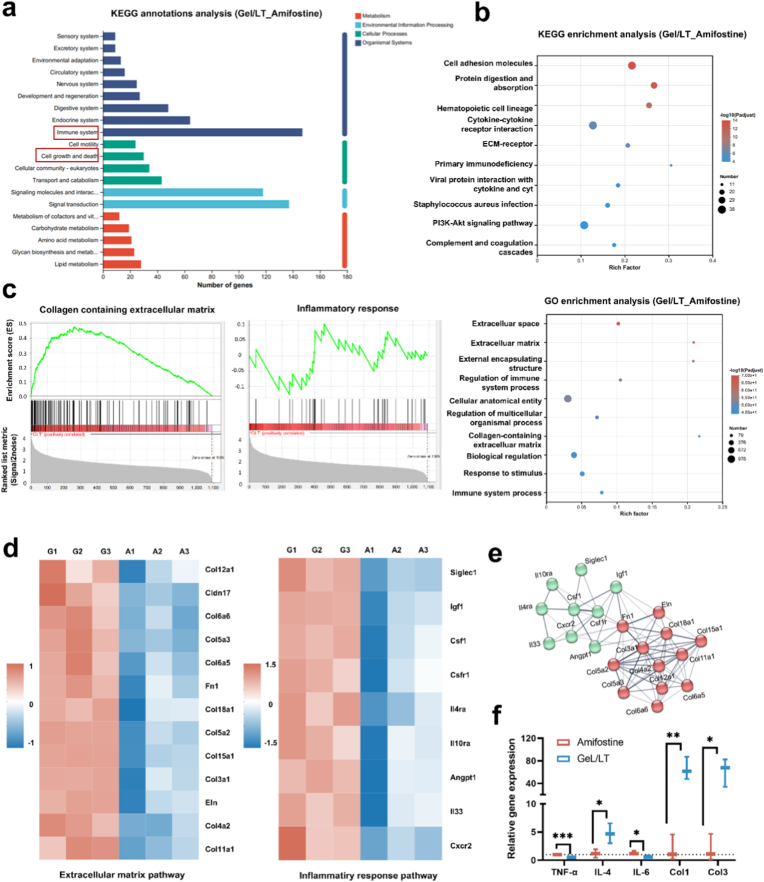
Transcriptome analysis of Gel/LT promoting RISI repair. (a) The KEGG annotations analysis and (b) Upregulated pathway in enriched KEGG and GO term of Gel/LT vs amifostine. (c) Gene set enrichment analysis (GSEA) analysis and (d) heatmap of the regulation of collagen containing extracellular matrix and inflammatory response. (e) String interaction network represents potential interaction relationships. (f) q-PCR validation for representative genes. *∗p* < 0.05, *∗∗p* < 0.01, *∗∗∗p* < 0.001.

**Fig. 6:**
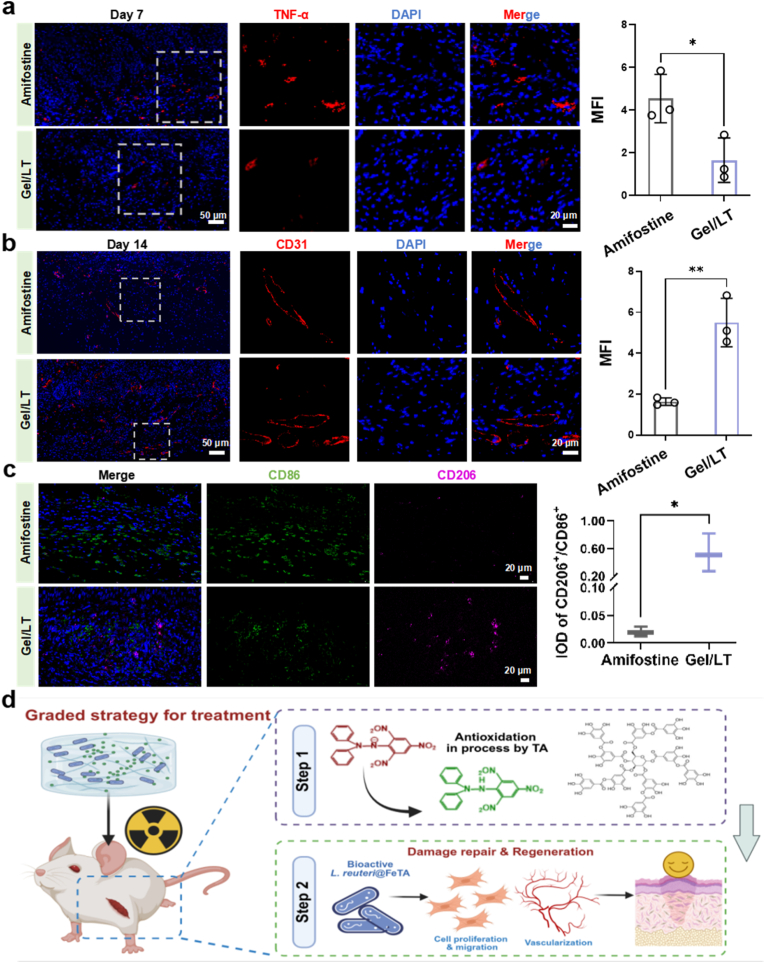
Gel/LT hierarchically regulates RISI healing. Immunostaining and quantification of (a) TNF-α at Day 7 and (b) CD31 at Day 14. (c) Immunostaining staining and quantification of M1/M2 macrophages (d) Hierarchical mechanisms of action of Gel/LT in RISI repair. ∗*p* < 0.05, and ∗∗*p* < 0.01.

RNA-seq was employed to explore the molecular mechanisms and intrinsic relationships underlying the superiority of Gel/LT hydrogels compared to commercial amifostine. Distinct transcriptome profiles were observed between the amifostine and Gel/LT groups (Fig. S10a). In total, the Gel/LT group exhibited 1090 upregulated genes and 915 downregulated genes when compared to those treated with amifostine, indicating that the hierarchical therapeutic strategy of Gel/LT, which first scavenges ROS and subsequently promotes tissue repair, plays a crucial role in the repair process following RISI (Fig. S10b–c). Additionally, the KEGG annotation analysis indicated that Gel/LT primarily upregulated immune-related signaling pathways associated with cellular processes such as cell growth and migration. This finding is consistent with previous cellular observations (Fig. 5a). KEGG and GO analyses revealed the enrichment of immune-related pathways (hematopoietic cell lineage, complement and coagulation cascades, regulation of immune system processes, immune system processes) and tissue repair-related pathways (cell adhesion molecules, cytokine-cytokine receptor interaction, ECM-receptor interaction, extracellular matrix, collagen-containing extracellular matrix) as illustrated in Fig. 5b. Consistently, gene set enrichment analysis (GSEA) revealed an upregulation in the collagen-containing extracellular matrix, as evidenced by the increased expression of *Fn1*, *Eln*, and collagen-related genes (*Col12a1*, *Col3a1*, *Col11a1*). In terms of immunoregulation, Gel/LT upregulated the expression of anti-inflammatory genes such as *Csf1*, *Igf1*, *Il4ra*, *Il10ra*, and *Il33*, demonstrating its exceptional ability to resolve inflammation, which may facilitate the transition of RISI wounds from the inflammatory phase to the proliferation and regeneration phases (Fig. 5c–e). Quantitative real-time polymerase chain reaction (q-PCR) was performed to verify the expression of key genes involved in these signaling pathways (Fig. 5f). Compared with the amifostine group, the Gel/LT group showed a significant downregulation in the expression of pro-inflammatory cytokines *Tnf-α* and *Il-6*, along with a marked upregulation in the anti-inflammatory cytokine *Il-4* and the collagen matrix genes collagen type I (*Col1*) and collagen type III (*Col3*), which was in accordance with the results of RNA sequencing. Immunostaining results at 7 and 14 days post-wound treatment validated this assumption. On day 7, pro-inflammatory cytokine TNF-α staining was conducted on wound tissues (Fig. 6a). It was observed that TNF-α secretion was minimal in the Gel/LT group; in contrast, the amifostine group exhibited intense red fluorescence at the wound site due to inadequate sustained ROS scavenging, indicating that the wound remained in a pro-inflammatory state. Immunofluorescence staining for CD31 was conducted to assess the extent of angiogenesis throughout the healing process. As illustrated in Fig. 6b, the Gel/LT group demonstrated the most pronounced CD31 staining in the wound areas on day 14, showing an increase of over two-fold compared to the amifostine groups. The immunofluorescence staining of macrophages (Fig. 6c), a key immune cell type involved in the wound repair process, further corroborate this result. In contrast to amifostine group, where skin tissues exhibited extensive infiltration of CD86^+^ M1 pro-inflammatory macrophages, the skin tissues of mice treated with Gel/LT showed a marked increase in CD206^+^ M2 pro-repairing macrophages, demonstrating that Gel/LT can effectively promote the polarization of macrophages toward the pro-repairing M2 phenotype *in vivo.* This finding indicates a favorable inflammatory response and more robust formation of granulation tissue. The RISI wound healing process encompasses intricate mechanisms and precise management, which include the rapid scavenging of ROS in the early stages and the facilitation of tissue repair in the later stages. Transcriptome and animal experimental results demonstrated that the hierarchical therapeutic strategy of Gel/LT facilitated ROS scavenging within 7 days of the onset of RISI through TA. This approach downregulated the expression of pro-inflammatory factors, such as TNF-α, while simultaneously upregulating the expression of anti-inflammatory factors, including IL-4 and Csf-1. These actions collectively facilitated the transition of wound repair from the immune phase to the proliferative phase. In the later stages of RISI repair, the released probiotics further promoted wound healing, resulting in enhanced formation of neo-collagen tissue and neovascularization at the wound site. Ultimately, this strategy demonstrated superior repair efficacy compared to the commercial drug amifostine (Fig. 6d). Therefore, Gel/LT exhibits significant clinical application potential in the field of RISI repair. And their core advantages lie in scalable fabrication and optimized administration routes, while also aligning with clinical repair requirements and providing strong support for their clinical translation.

## 4 Conclusion

In summary, a multi-activity hydrogel with a time-sequenced response has been developed to address the various stages of RISI's complex lesion environment. The hydrogel's well-designed structure facilitates effective loading and delivery performance, while the release of TA aids in the removal of excess free radicals during the initial stage. Additionally, the living strains within the hydrogel respond to the normal wound microenvironment and are released at the wound site, exerting biological properties such as angiogenesis and tissue repair. Through the RISI model *in vivo*, we found that the therapeutic effect of hydrogel significantly surpassed that of traditional amifostine therapy. Results from transcriptomics revealed its molecular mechanism, which involves reducing ROS and promoting the regeneration of extracellular matrix components, such as collagen, through hierarchical regulation. This study demonstrates a novel therapy that achieves sequential treatment by responding to the microenvironment at different stages of RISI.

## CRediT authorship contribution statement

**Xiaowen Han:** Investigation, Data curation, Conceptualization. **Chen Zhou:** Methodology, Investigation, Data curation. **Ruiling Xu:** Investigation. **Zhimin Jia:** Investigation. **Ying Liu:** Resources. **Shan Chen:** Software. **Wei Tang:** Data curation. **Xiaoan Li:** Writing – review & editing, Writing – original draft. **Liangxue Zhou:** Writing – review & editing, Writing – original draft. **Yong Sun:** Writing – review & editing, Writing – original draft, Supervision, Funding acquisition.

## Declaration of competing interest

The authors declare that they have no known competing financial interests or personal relationships that could have appeared to influence the work reported in this paper.

## Data Availability

Data will be made available on request.
