# Supplementary material for: Functionalized hydrogel sequentially deliver tannic acid and bioactive probiotics for radiation-induced skin injury
Source: Mater Today Bio. 2025 Dec 31;36:102753. doi: 10.1016/j.mtbio.2025.102753 (PMC12810556; doi:10.1016/j.mtbio.2025.102753)
Supplement: Multimedia component 1 [file mmc1.docx]

**Supporting Information for**

**Functionalized Hydrogel Sequentially Deliver Tannic Acid and Bioactive Probiotics for Radiation-Induced Skin Injury**

Xiaowen Han ^a,1^, Chen Zhou ^b,1^, Ruiling Xu ^a^, Zhimin Jia ^c^, Ying Liu ^d^, Shan Chen ^d^, Wei Tang ^d^, Xiaoan Li ^a*^, Liangxue Zhou ^e,*^, Yong Sun ^b,*^

**a.** NHC Key Laboratory of Nuclear Technology Medical Transformation, Mianyang Central Hospital, School of Medicine, University of Electronic Science and Technology of China, Mianyang, 621000, China

**b.** National Engineering Research Center for Biomaterials, Sichuan University, 29 Wangjiang Road, Chengdu, Sichuan, 610064, PR China

**c.** Department of Nuclear Medicine, Mianyang Central Hospital, Mianyang, China

**d.** Institute of Materials, China Academy of Engineering Physics, Jiangyou, 621907, China

**e.** Sichuan Provincial Engineering Research Center of Nuclear Medical Equipment Translation and Application, Mianyang Central Hospital, School of Medicine, University of Electronic Science and Technology of China, Mianyang, 621000, China.

**1.** These authors contributed equally to this work.

**Results and discussion**


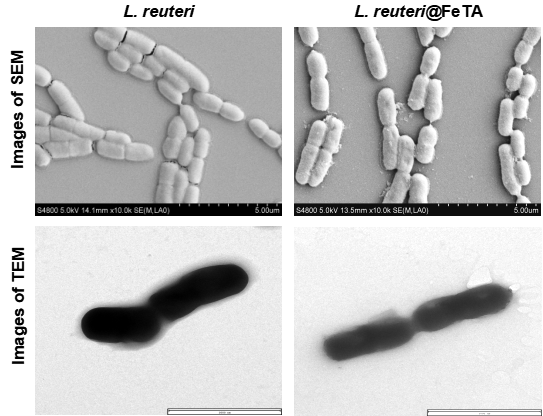


**Fig. S1.** Representative SEM and TEM images of *L. reuteri* and *L. reuteri*@FeTA


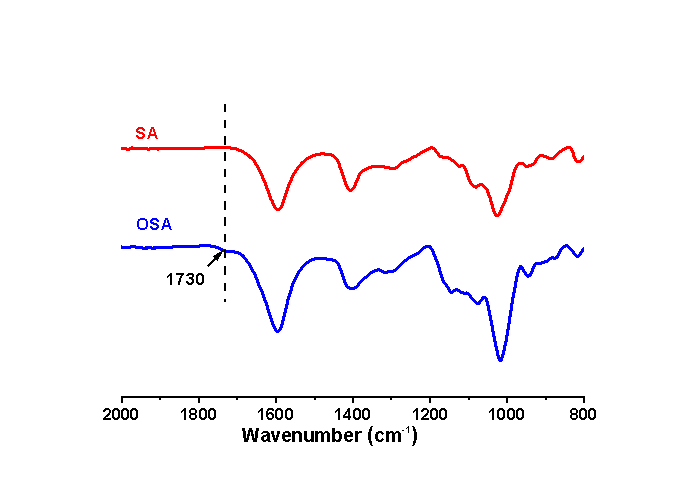


**Fig. S2.** The ATR-FTIR spectrum of SA and OSA.


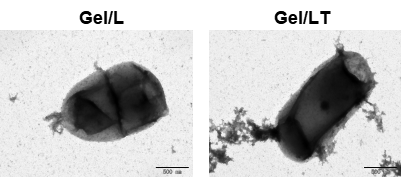


**Fig. S3.** Representative TEM images of *L. reuteri*@FeTA in Gel/L and Gel/LT.

**
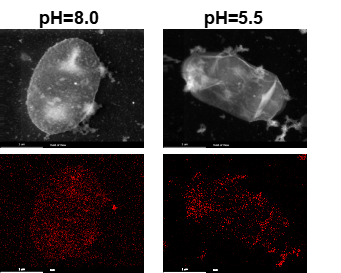
**

**Fig. S4.** The Fe element maps (b) on *L. reuteri*@FeTA in Gel/LT at different pH value.


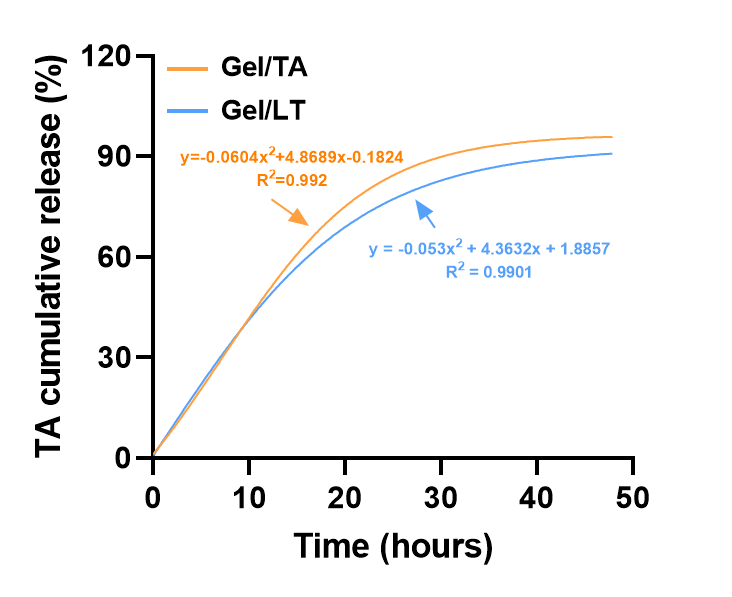


**Fig. S5.** The release kinetics fitting curve of TA in hydrogel.


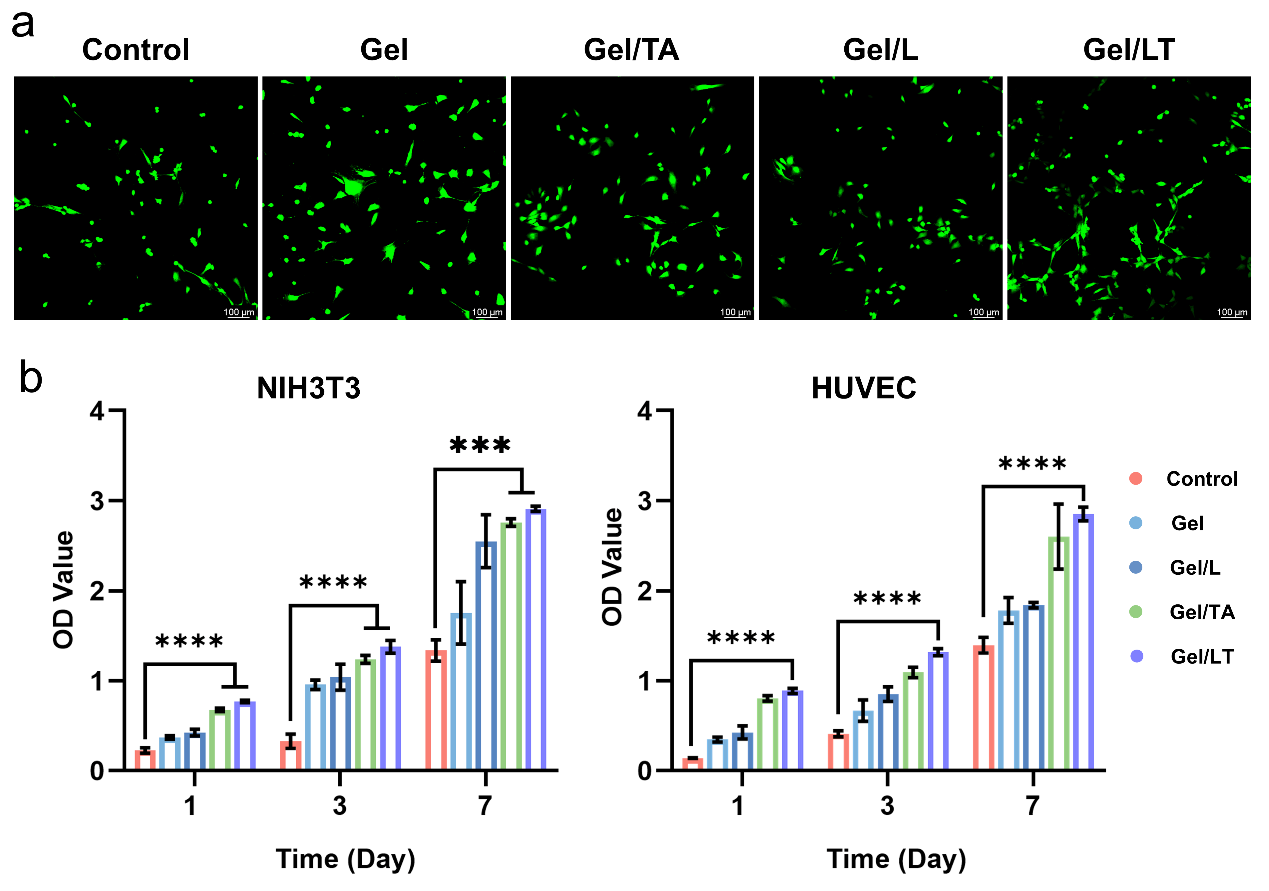


**Fig. S6.** Cytocompatibility of Gel/LT on fibroblasts and endothelial cells. (a) Live/dead staining of NIH3T3 cells (scale bar: 100 μm); (b) CCK-8 assays of NIH3T3 and HUVECs. ****p* < 0.001, and *****p* < 0.0001.


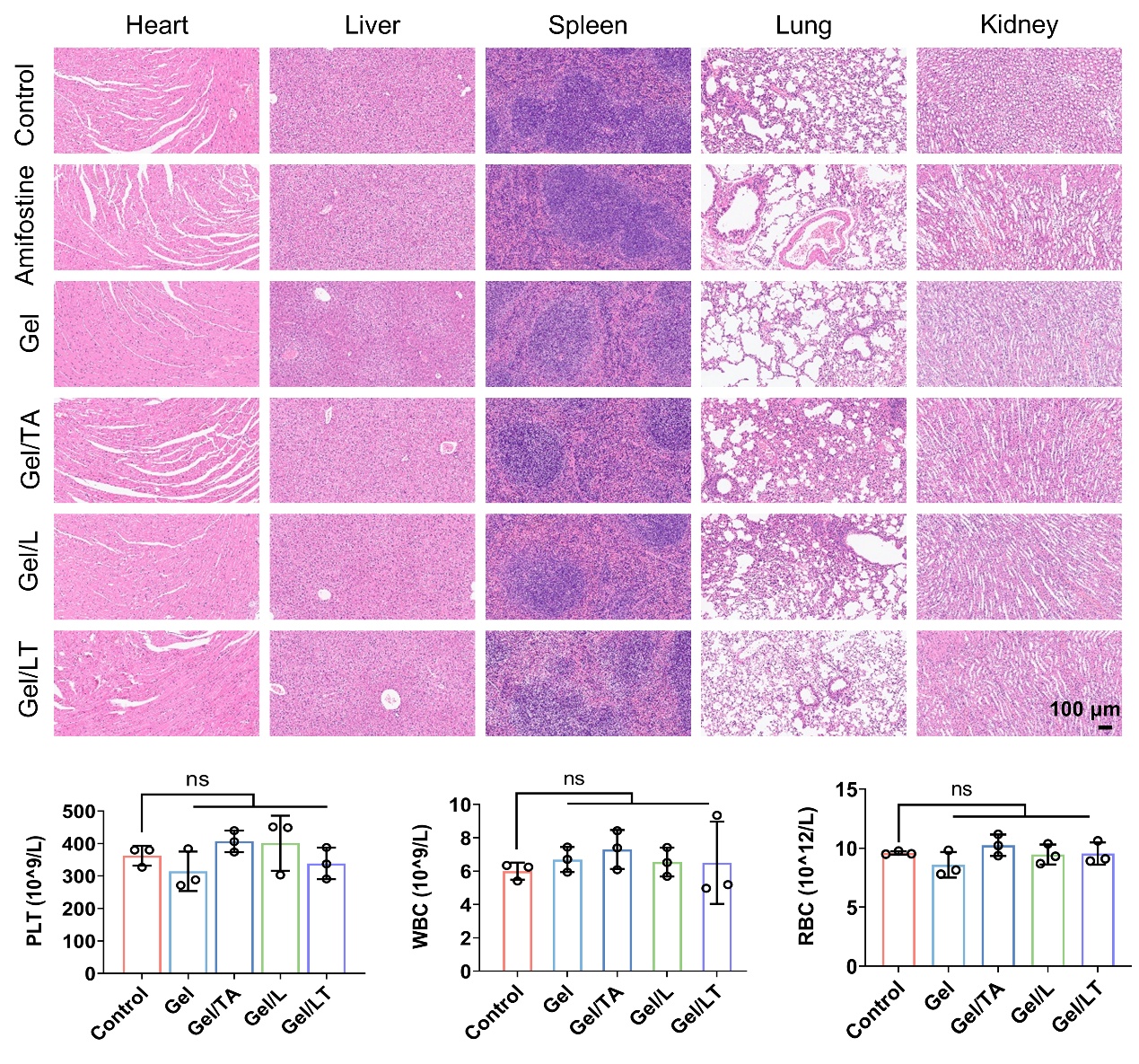


**Fig. S7.** Biosafety of Gel/LT (scale bar: 100 μm).


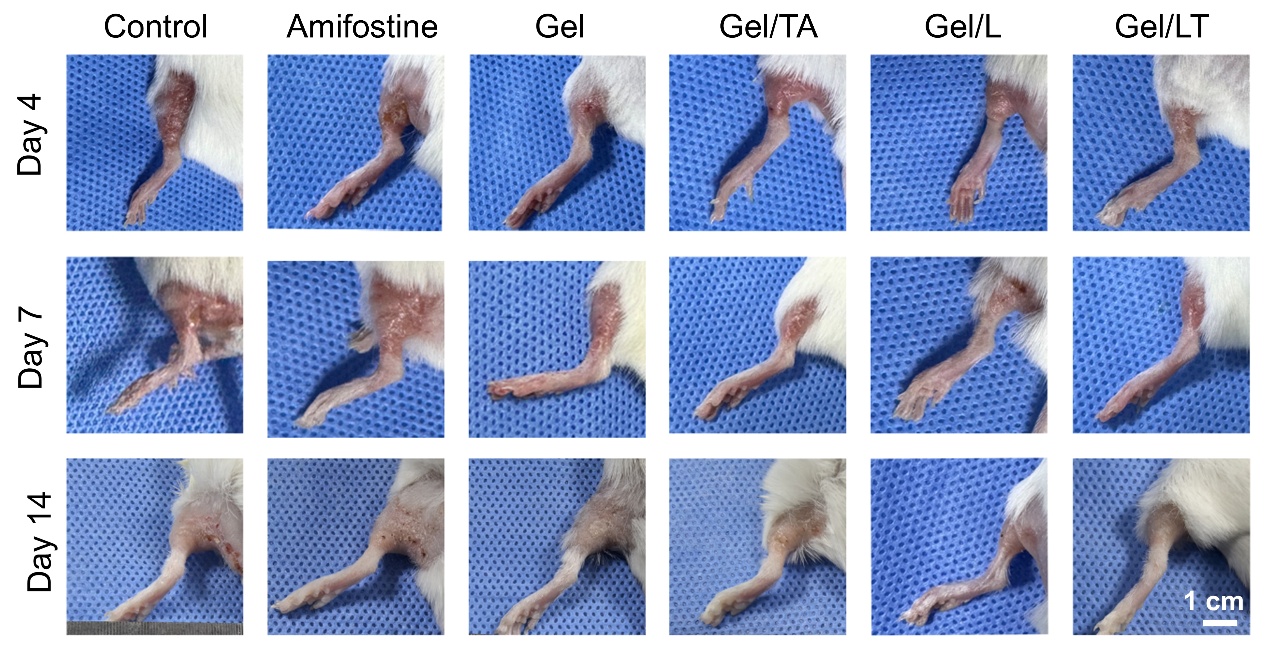


**Fig. S8.** Representative images of skin injury area under different treatment.


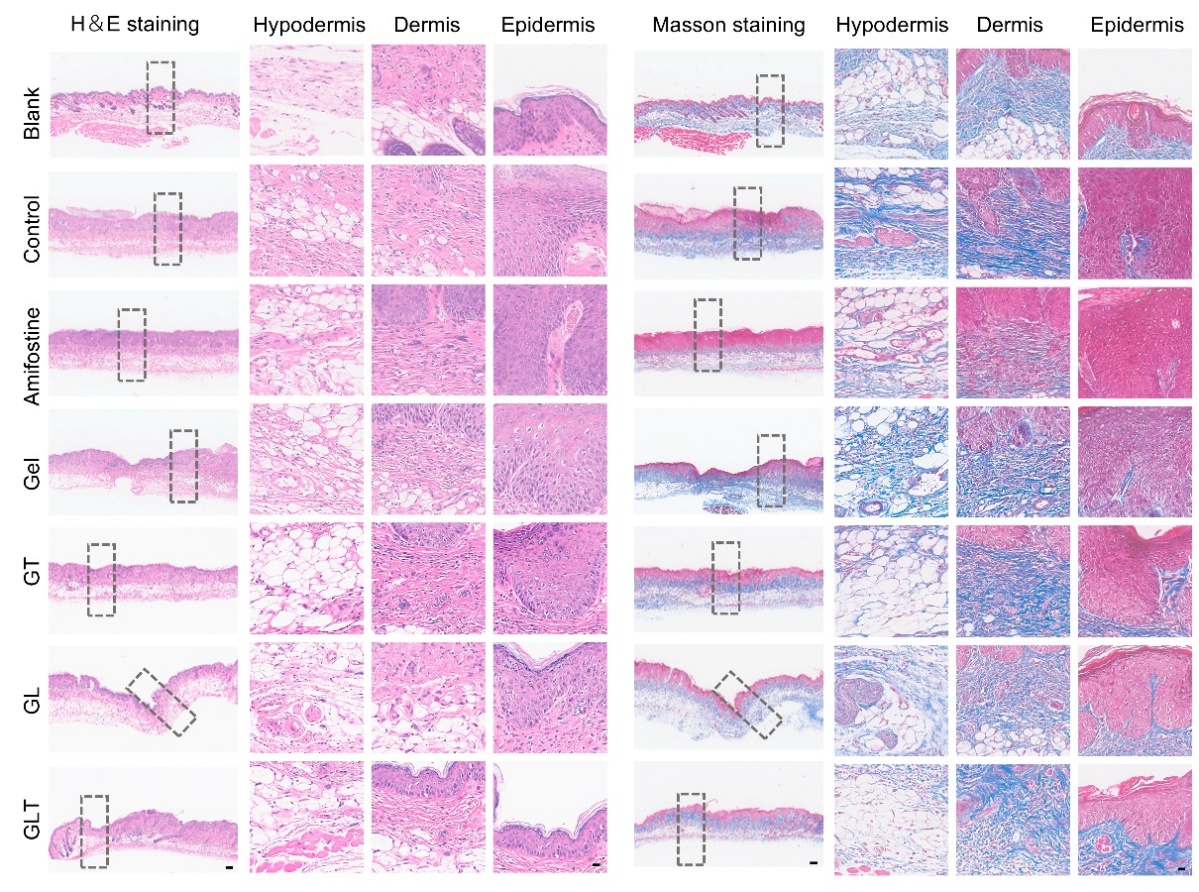


**Fig. S9.** H&E and Masson staining of wound area ((scale bar: 50 μm (left) and 20 μm (right)).


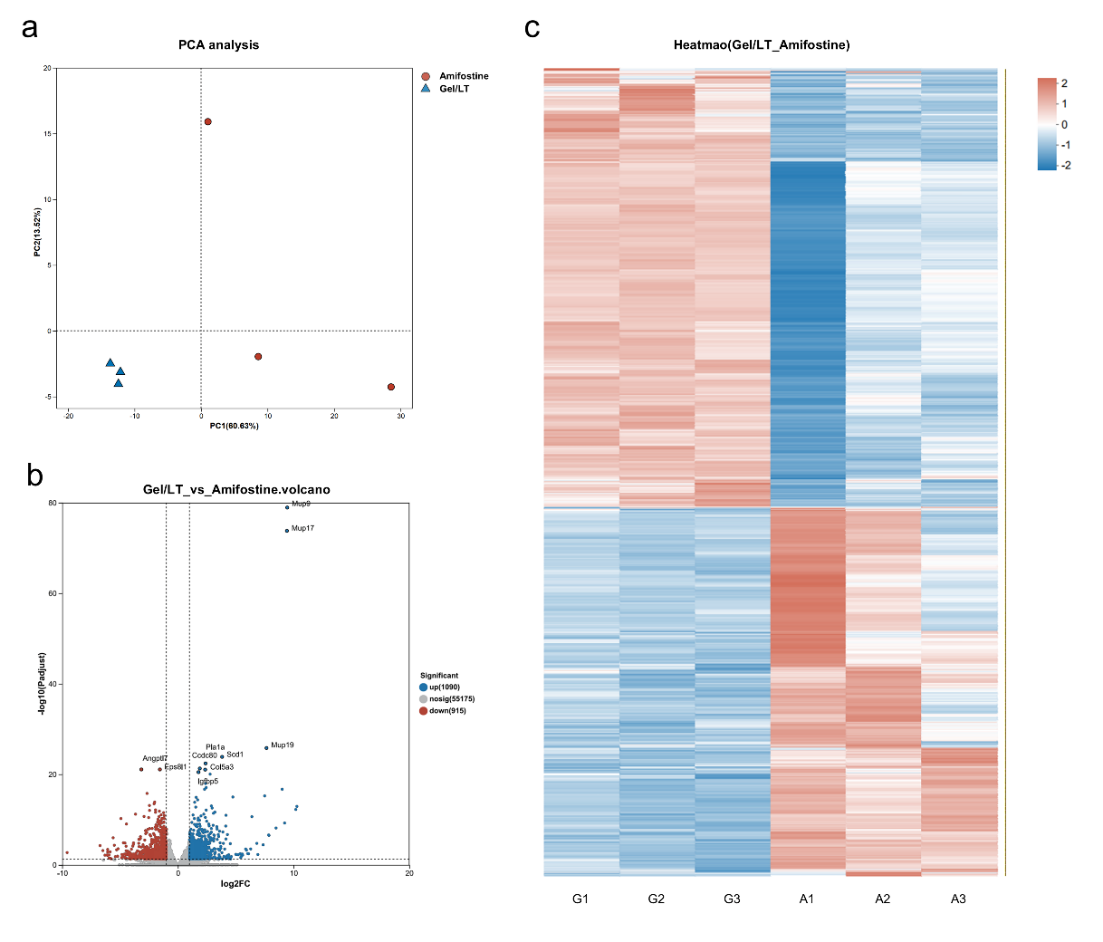


**Fig. S10.** Transcriptome analysis of Gel/LT vs amifostine. (a) PCA analysis. (b) volcano and (c) heatmap of Gel/LT vs amifostine
